# Dragons guard secrets: integrative systematics of *Pteraeolidia semperi* species complex (Gastropoda, Nudibranchia) from Vietnam

**DOI:** 10.3897/zookeys.1293.195366

**Published:** 2026-10-02

**Authors:** Maria N. Meshik, Tatiana I. Antokhina, Yury V. Deart, Olga V. Chichvarkhina, Irina A. Ekimova

**Affiliations:** 1 Invertebrate Zoology Department, Lomonosov Moscow State University, Leninskie Gory 1-12, Moscow, 119234, Russia Invertebrate Zoology Department, Lomonosov Moscow State University Moscow Russia; 2 Laboratory of Marine Invertebrate Morphology and Ecology, A.N. Severtsov Institute of Ecology and Evolution RAS, Leninsky Prospekt 33, Moscow, 119071, Russia Laboratory of Marine Invertebrate Morphology and Ecology, A.N. Severtsov Institute of Ecology and Evolution RAS Moscow Russia; 3 National Scientific Center of Marine Biology FEB RAS, Vladivostok, Russia National Scientific Center of Marine Biology FEB RAS Vladivostok Russia

**Keywords:** Cryptic diversity, molecular phylogeny, Nudibranchia, photosymbiosis, phylogeography, Vietnam

## Abstract

The nudibranch genus *Pteraeolidia* has traditionally been treated as taxonomically simple, but recent molecular studies suggest that it includes hidden species diversity across the Indo-Pacific. In this study, the *Pteraeolidia semperi* species complex was investigated from the coastal waters of Vietnam using an integrative approach. Thirty-five specimens from five localities were analysed using mitochondrial (COI, 16S rRNA) and nuclear (H3) markers, together with external and internal morphological data and haplotype network analysis. Our results revealed two deeply divergent clades (Clades A and B) occurring sympatrically in Vietnam. These clades correspond to several molecular operational taxonomic units and show strong geographic structure across the Indo-West Pacific. One of these units (MOTU20), which falls within Clade B, corresponds to *Pteraeolidia ianthina* (Angas, 1864), a valid species restricted to its type locality, Sydney Harbour, New South Wales; it is treated here as separate from the *P. semperi* complex. Nuclear data support the separation of the two clades as independent evolutionary lineages, whereas relationships among mitochondrial lineages within each clade remain unresolved. The two clades also differ in external morphology, especially in body colouration, pigmentation pattern, and ceratal arrangement, while internal anatomy is largely similar. Mitochondrial divergence in *Pteraeolidia* is exceptionally high, including within clades, and exceeds values usually reported between species. This unusual pattern may reflect accelerated mitochondrial evolution, possibly linked to the photosymbiotic lifestyle of these nudibranchs. Our data show that Vietnamese populations of *Pteraeolidia* include at least two species within a broader and highly diverse species complex. These findings confirm that *Pteraeolidia semperi* represents a complex of cryptic or semi-cryptic taxa and highlight the need for genomic data to resolve species limits more precisely.

## Introduction

*Pteraeolidia* Bergh, 1875 is a remarkable and widespread genus of nudibranch molluscs, informally known as the Blue Dragon. Its range extends from the southwestern Indian Ocean off the coast of South Africa, up to the Red Sea and west to the Coral Triangle, reaching the Hawaiian Islands in the east, Japan in the north and eastern Australia in the south (Wilson and Burghardt 2015). *Pteraeolidia* belongs to the species-rich family Facelinidae of the suborder Aeolidina. Until recently, this genus was believed to be represented by only one species: a revision conducted by Rudman (1982) designated *P. ianthina* (Angas, 1864) as the single distinct species, and *P. semperi* (Bergh, 1870) and *P. scolopendrella* Risbec, 1928 were considered its junior subjective synonyms. However, recent studies have identified hidden genetic diversity within the genus *Pteraeolidia* with two or more distinct genetic groups (Yorifuji et al. 2012; Wilson and Burghardt 2015; Soon et al. 2023). The existence of hidden species diversity is further indicated by a wide range of external morphological features, including colouration, as well as a diverse range of habitats and ecological adaptations.

Wilson and Burghardt (2015) established that *P. ianthina* is restricted to its type locality, Sydney Harbour, New South Wales, and is not found in other regions. *Pteraeolidia semperi* and *Pteraeolidia scolopendrella* are species complexes requiring further revision. The hidden diversity within the *P. semperi* species complex was also confirmed in a subsequent work by Soon et al. (2023) who demonstrated that, based on molecular analysis, the genus *Pteraeolidia* is divided into two large clades. Furthermore, elevated levels of mitochondrial DNA variability were observed in specimens belonging to one of the two clades. The nature of this phenomenon is not fully understood, and the morphological differences between the two clades were not identified.

Representatives of *Pteraeolidia* have several unique features that set them apart from other aeolid taxa. Among them are long body (individuals can reach 300 mm; see Hoover 2006), caring for offspring (Rose and Hoegh-Guldberg 1982), as well as a highly specialised symbiosis with zooxanthellae (Rudman 1982). Zooxanthellae are symbiotic algae belonging to the phylum Dinoflagellata, family Symbiodiniaceae (LaJeunesse 2020). Nudibranchs acquire symbionts through horizontal transmission from food containing zooxanthellae (Wecker et al. 2015). Acquired zooxanthellae are taken up by the digestive gland cells, where they subsequently live, photosynthesise and multiply intracellularly (Hoegh-Guldberg and Hinde 1986; Hoegh-Guldberg et al. 1986). The colouration of individuals from the genus *Pteraeolidia* greatly depends on the presence and concentration of zooxanthellae (Hoegh-Guldberg and Hinde 1986) as *Pteraeolidia* juveniles lacking zooxanthellae are nearly completely white.

Symbiotic relationships with zooxanthellae (Symbiodiniaceae) have been observed among various nudibranch groups (Burghardt et al. 2008) performing independent evolution of nudibranch-zooxanthellae symbioses (Rola et al. 2022). Particularly, *Pteraeolidia* has highly specialised morphological adaptations that enhance their photosynthetic efficiency (Rudman 1982). These adaptations include multiple branching of the digestive gland into fine gastrovascular canals, the intersection of terminal gastrovascular canals of the hemocoel and their positioning in close proximity to the body wall (Vorobyeva et al. 2023), thinning of the epidermal layer of cerata (Goodheart et al. 2018), and the arrangement of cerata in a fan-like pattern on basal outgrowths (Togawa et al. 2019). Although *Pteraeolidia* is frequently considered an example of a highly specialised photosymbiotic system useful for model studies, there is still a limited understanding of metabolic relationships between the nudibranchs and their symbionts (Mizobata et al. 2023). Also, symbiont biodiversity across various *Pteraeolidia* lineages remains poorly explored across its broad distribution range: a single study has shown the lack of symbiont specificity within two sympatric *Pteraeolidia* clades in Singapore (Soon et al. 2023).

In this paper we present the first integrative analysis of *P. semperi* from Vietnam coastal waters. The nudibranch fauna of Vietnam remains poorly studied; a single review of its biodiversity identified approximately 120 sea slug species (Martynov and Korshunova 2012), which is much less than the nudibranch fauna of adjacent Indo-West Pacific waters (Gosliner et al. 2018). Recent DNA-based taxonomical studies identified the presence of hidden diversity within many nudibranch groups including Aeolidina in Vietnam (Martynov and Korshunova 2012; Ekimova et al. 2019, 2022; Grishina et al. 2023), suggesting similarly high genetic and morphological diversities could also be observed within *Pteraeolidia* populations. The specific aims of this study are to conduct the first integrative taxonomic analysis of the *P. semperi* species complex from the coastal waters of Vietnam, using molecular (COI, 16S rRNA, H3), phylogenetic, and morphological data to reveal hidden diversity, delimit species boundaries, and assess population structures across collection sites.

## Materials and methods

### Collection data

**Figure 1.**
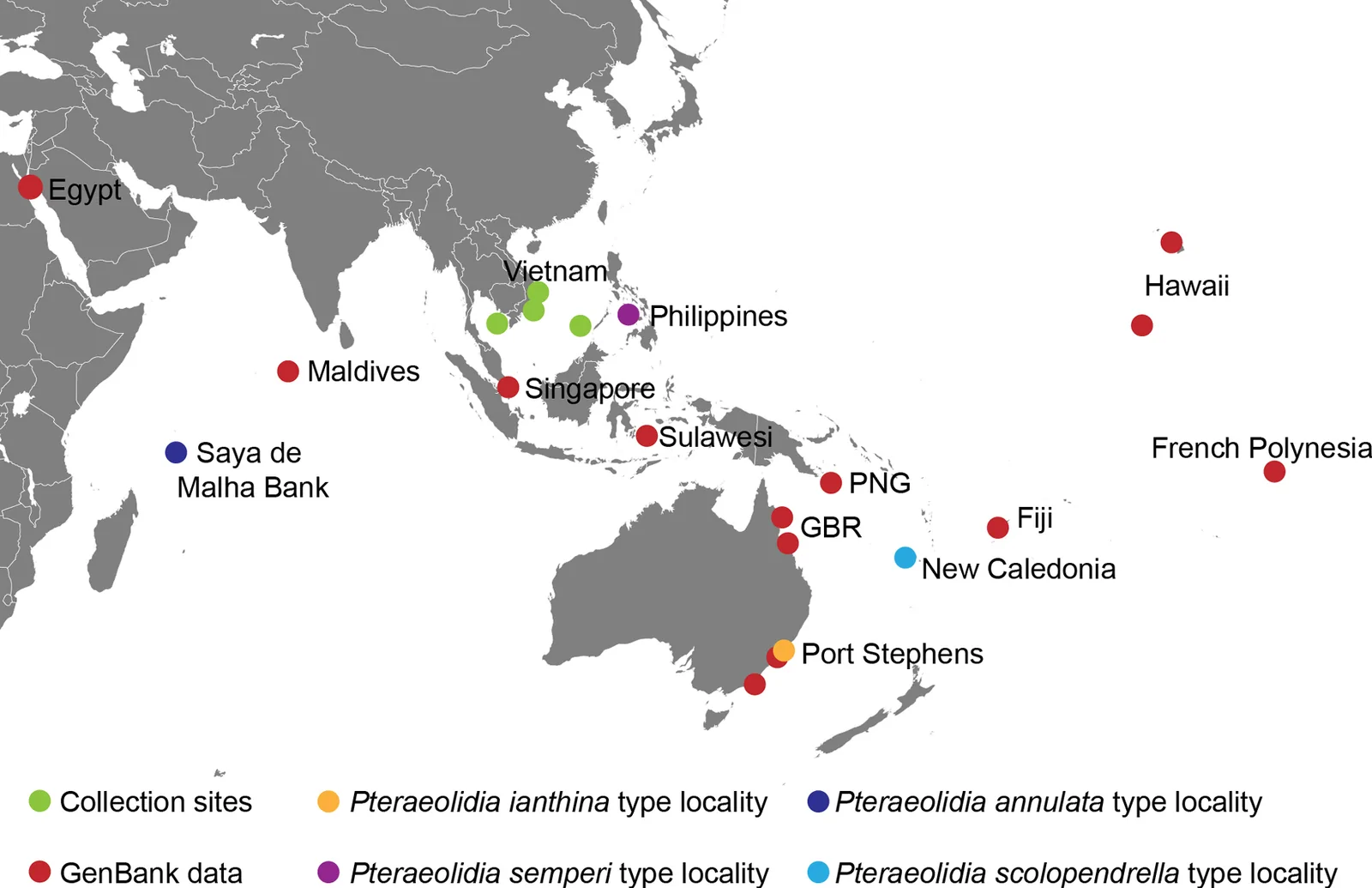
Map of the Indo-West Pacific representing collection sites of *Pteraeolidia* specimens studied herein and the localities of GenBank records, shown in different colours. The type locality of each species is also indicated, based on the original species descriptions (no genetic data are available from the type localities).

A total of 35 specimens were collected during expeditions of the Vietnamese-Russian Tropical Centre in 2016–2023 in five localities of central and southern Vietnam: Nha Trang, Phan Rang, Binh Dinh, Tho Chu, and the Spratly Islands (Fig. 1). All samples were collected by scuba diving at depths of 2–16 m. All specimens were photographed and fixed in 96% ethanol for morphological and molecular studies. Voucher specimens are deposited in the collections of the National Scientific Centre of Marine Biology, Far Eastern Branch RAS. Details of voucher numbers for each specimen and sampling localities are given in Suppl. material 1.

### DNA extraction, amplification, sequencing

DNA was extracted from a small piece (~ 1 mm^3^) of foot muscle tissue using a PALL™ AcroPrep 96-well purification plate by PALL Corp., following the outlined protocol by Ivanova et al. (2006). Obtained DNA samples were used as a template for amplification of partial cytochrome c oxidase subunit I (COI), 16S rRNA, and histone H3. Amplification conditions and primers used for amplification and sequencing are presented in Suppl. material 2. The reaction mixture composition for amplification (total volume 25 μL): 5 μL of 5 × Taq Red buffer (Eurogen Lab), 0.5 μL dNTPs (Eurogen Lab), 0.5 μl HS Taq DNA Polymerase (Eurogen Lab), 0.3 μL mix of forward and reverse primers (10 μM stock), 1 μL genomic DNA, and 17.7 μL deionised water (MilliQ). The reaction products were analysed by electrophoresis on a 1.5% agarose gel using a 1 × TAE buffer. The same primers were used for both the forward and reverse sequencing reactions. Sequencing was carried out using the Applied Biosystems 3500 genetic analyser (Life Technologies, USA) and the NANOPOR-05 sequencer (Syntol, Russia), both located at the Koltsov Institute of Developmental Biology RAS. NovaDye v. 3.1. reagent kit was used for sequencing according to the manufacturer’s protocol.

### Data processing

All new sequences were deposited at the GenBank public database (Suppl. material 1). Raw reads for each gene were assembled and quality-controlled to ensure accurate base-calling. Any sites with potential errors were further investigated and corrected using Geneious v. 10.0.9 (Biomatters, Auckland, New Zealand). Original data and publicly available sequences were aligned using MAFFT 7 web service (Katoh et al. 2019). To detect pseudogenes, the protein-coding gene sequences (COI and H3 fragments) were translated into amino acid sequences using the Geneious v. 10.0.9 software. All sequences were additionally checked by BLAST against GenBank; the closest matches were to aeolid gastropods, with no hits on Symbiodiniaceae or other dinoflagellates. The final alignments were of 671 bp for COI, 471 bp for 16S, and 338 bp for H3.

### Species delimitation analyses

During the study, we employed three methods for analysing species boundaries based on molecular genetic features: comparison of topologies in molecular phylogenetic trees, comparison of *p*-distances, and use of the ASAP (Assemble Species by Automatic Partitioning) method. *P*-distances were calculated using the Geneious v. 10.0.9 software. For the ASAP method (Puillandre et al. 2021), a program available on an online resource (https://bioinfo.mnhn.fr/abi/public/asap/) was utilised. The analysis employed three proposed models: Jukes-Cantor (JC69), Kimura 2-parameter (K80), and simple pairwise *p*-distance. In addition to the combined dataset, ASAP was run separately for each of the two main clades (Clade A and Clade B) under the same three models, to test whether the strongly heterogeneous divergence across the dataset influenced the estimation of the barcode gap and the resulting partition.

As an additional, nuclear line of evidence for species boundaries, the aligned H3 sequences were examined for fixed (diagnostic) nucleotide differences between the two sympatric clades—that is, substitutions present in all members of one clade and absent in all members of the other. Alignments were inspected visually in Geneious v. 10.0.9, and the number of substitutions and pairwise *p*-distances between clades were recorded. Because H3 is a biparentally inherited nuclear marker, the presence of substitutions that are fixed between sympatric clades indicates restricted gene flow, and hence reproductive isolation, whereas ongoing interbreeding would be expected to homogenise the nuclear genome and prevent such fixed differences from accumulating.

### Phylogenetic analyses

Phylogenetic analysis was conducted for each fragment individually. The most appropriate nucleotide evolution model was selected using the Modeltest-NG toolkit (Darriba et al. 2020), based on the Bayesian information criterion (BIC), for each partition. For the COI partition, the best-fitting model was TVM + I + G. For 16S, the model TPM3uf + I + G was chosen. For H3, the GTR + G + I model provided the best fit. Phylogenetic reconstruction was performed using Bayesian estimation of posterior probabilities and maximum likelihood-based phylogenetic inference. Bayesian estimation was carried out in MrBayes v. 3.2.6 (Ronquist and Huelsenbeck 2003) applying evolutionary models appropriate for the software. Models were selected for each partition in MEGA X (v. 10) (Kumar et al. 2018) under the BIC; the best-fitting schemes were GTR+G+I for COI, GTR+G for 16S, and GTR+G+I for H3, and these were applied in MrBayes. The analysis was run with default settings. A random starting tree was used, and the analysis run for 10^7^ generations. Maximum likelihood inference was performed in RAxML v. 8.2.12 (Stamatakis 2014) using the HPC-PTHREADS-AVX option, with 1000 pseudoreplicates, under the GTRCAT model of nucleotide evolution. Final phylogenetic tree images were rendered in FigTree Drawing Tool v. 1.4.4. and further modified in Adobe Illustrator CC 2015. Posterior probability values (PP) greater than 0.99, and bootstrap values (BS) above 90%, indicate strong support for a particular hypothesis. Values between 0.95 and 0.98 for PP, and between 75% and 89% for BS, indicate moderate support. Values below 0.9 (PP) and 60% (BS) indicate weak support. Branches with lower levels of support are considered to be unsubstantiated (Ekimova et al. 2021).

### Haplotype networks

The population study was based on COI sequence data, including both original sequences and those obtained from GenBank (Suppl. material 3). Haplotype networks were generated using PopArt software (Leigh and Bryant 2015), with the TCS network method (Clement et al. 2002) to construct networks. The final networks were then edited using Adobe Illustrator CC 2015 software.

### Morphological studies

For the analysis of living colouration and external morphological features, photographs of living adult and juvenile *Pteraeolidia* molluscs in their habitat and in a petri dish with black background were used. Juvenile specimens were distinguished not by a fixed size threshold but by two qualitative characters: the near-absence of zooxanthellae, reflected in their pale, whitish colouration, and the arrangement of cerata in a single row rather than in clusters, the latter being characteristic of adults.

Attention was paid to the peculiarities of body colouration, oral tentacles, and rhinophores, as well as the number, location, colouration, and arrangement of cerata. Internal morphology was investigated by examining the morphology of the feeding apparatus, specifically the radula and jaw. The buccal masses of each specimen were extracted and soaked in 10% sodium hypochlorite solution (bleach) to dissolve soft tissue. The radulae and jaws were then rinsed with distilled water. They were mounted on aluminium stubs, air-dried and coated with a mixture of gold and platinum. The features of the jaws and radulae were analysed using scanning electron microscopy with Quattro S (Thermo Scientific, USA) and JEOL JSM-6380LA (JEOL, USA) microscopes.

## Results

### Molecular analyses

Species delimitation

**Figure 2.**
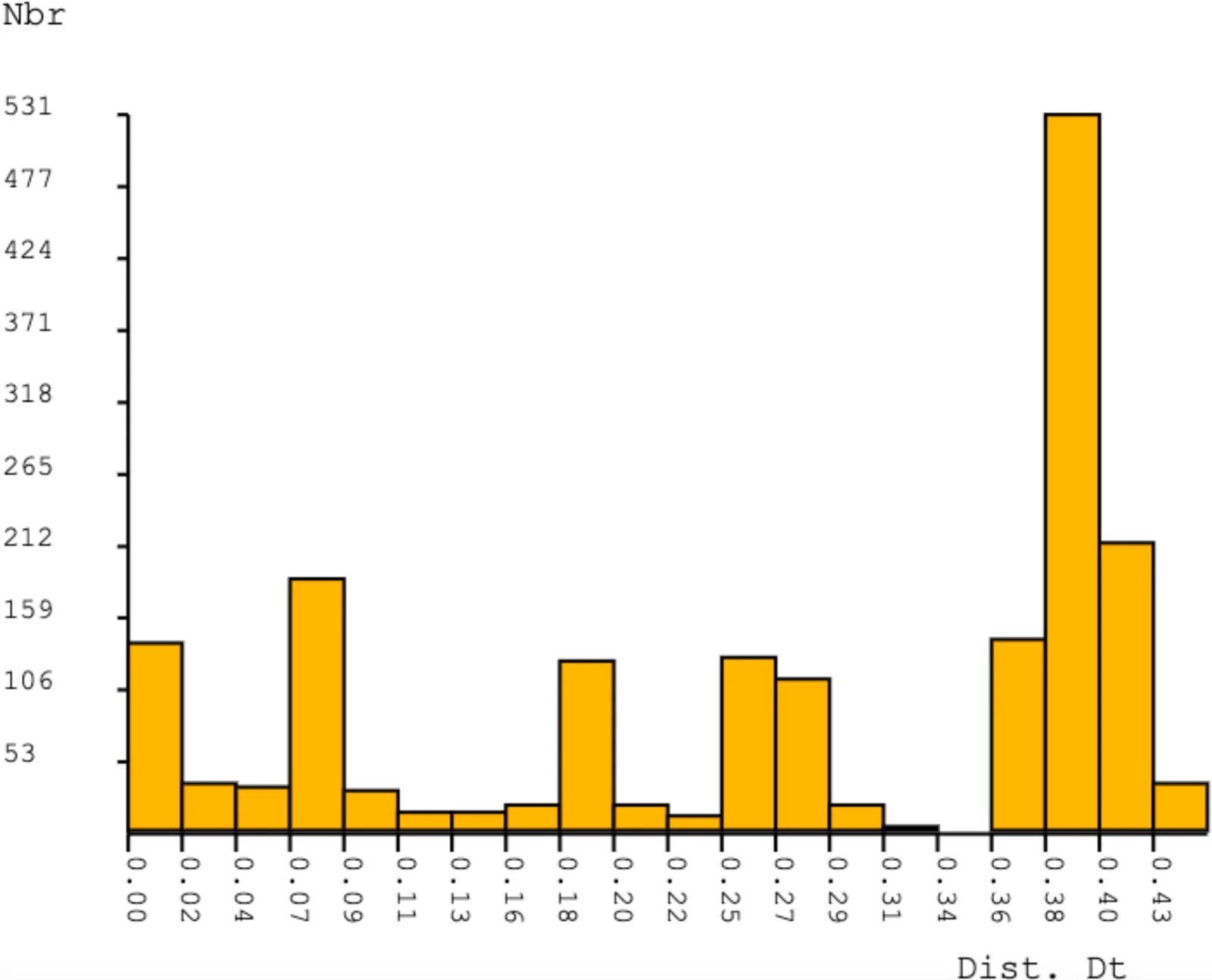
Histogram of genetic distances generated using the ASAP algorithm (Puillandre et al. 2021). The x-axis shows the pairwise genetic distance between two sequences (computed under the JC69 model), and the y-axis shows the frequency, i.e., the number of sequence pairs falling within each distance interval. The barcode gap is the trough separating the cluster of smaller (putatively intraspecific) distances from that of larger (putatively interspecific) distances; in this dataset several such gaps are present (see text), which underlies the ambiguity in the number of delimited MOTUs.

The ASAP method has been developed for the specific purpose of delimitation analysis and operates by identifying the barcode gap, which represents the disparity between intraspecies and interspecies mutations (Puillandre et al. 2021). However, in contrast to the conventional view, the analysis revealed the presence of four distinct barcode gaps (Fig. 2). This finding has implications for the estimation of species numbers, which can range from six, nine to 19 or 20, depending on the value of asap-score, which are not markedly disparate from one another. To facilitate further interpretation of the results, the samples were categorised into groups in accordance with the distribution proposed by the ASAP algorithm. The number of subsets corresponding to the minimum ASAP score value was selected: 20 units for a value of 4.5. The 20 subsets were designated as MOTU-20 (Molecular Operational Taxonomic Unit). The correspondence between sample numbers and MOTU numbers is indicated in Suppl. material 4. Specimens from Vietnam were united in four groups: MOTU1, MOTU4, MOTU5, and MOTU14. *P*-distances for the COI, 16S, and H3 markers are shown in Suppl. material 5. The 20-MOTU partition was recovered consistently across the three distance models (20 MOTUs under JC69 and simple *p*-distances, 21 under K80). When ASAP was run separately for each clade (Suppl. material 7), Clade A yielded the same 13 MOTUs as in the combined analysis, whereas Clade B was partitioned more finely (12 or 13 MOTUs versus 7 in the combined analysis). As this finer subdivision appeared only when Clade B was analysed in isolation, we regard it as an effect of the reduced distance distribution rather than evidence of additional species-level lineages.

#### Molecular phylogenetic analysis

**Figure 3.**
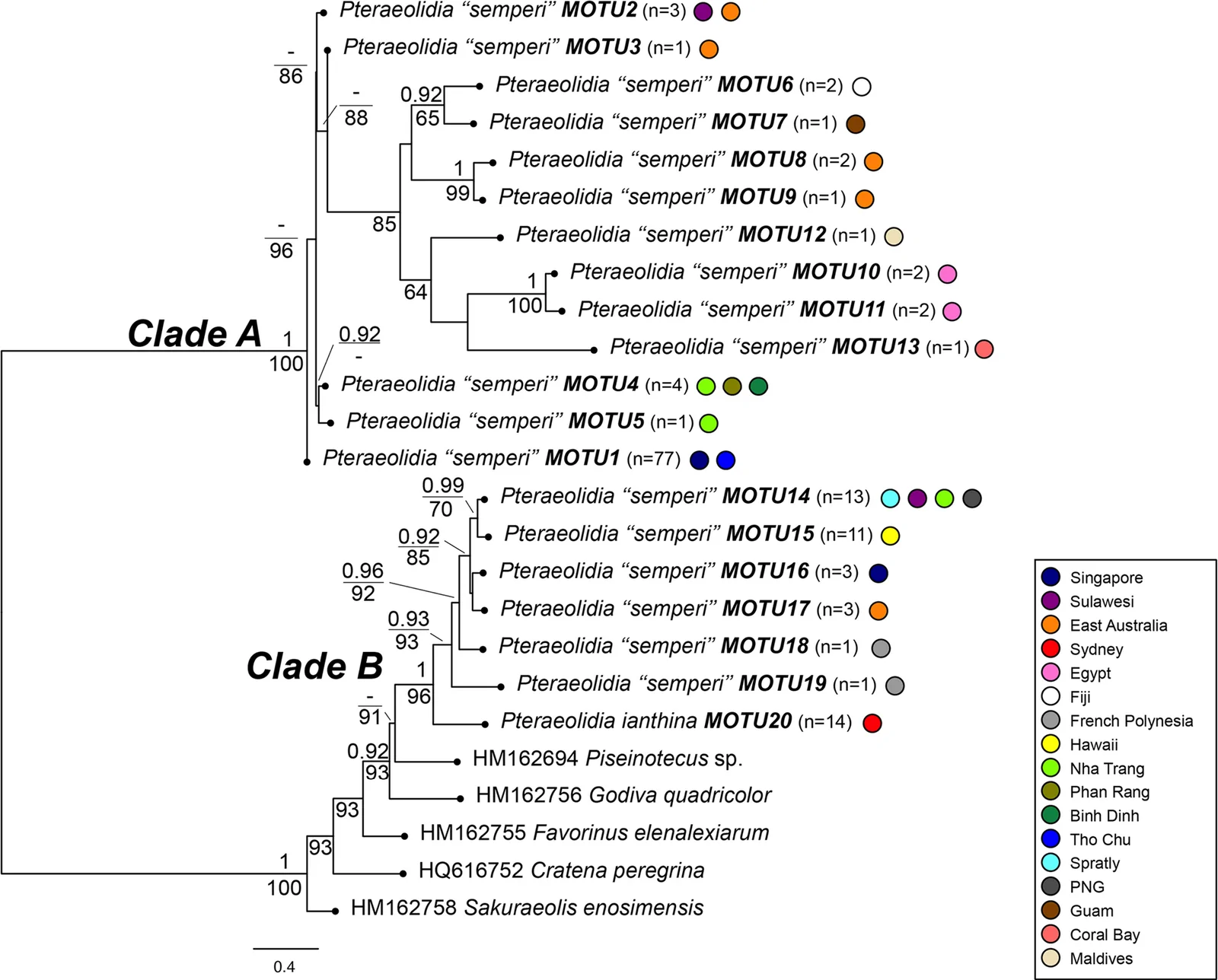
Maximum-likelihood phylogenetic tree of the *Pteraeolidia semperi* species complex and *P. ianthina* based on COI sequences, with specimens grouped into molecular operational taxonomic units (MOTUs) to provide a more concise representation. Coloured circles denote the sampling localities represented within each MOTU, as indicated in the legend. Numbers in parentheses after each MOTU name indicate the number of specimens included in that MOTU. Values above branches represent Bayesian posterior probabilities, whereas values below branches indicate bootstrap support values from the maximum-likelihood analysis. The five taxa at the base of the tree were used as outgroups.

Phylogenetic reconstructions based on the COI marker, constructed using the maximum-likelihood (ML) and Bayesian-inference (BI) methods produced similar topologies. The samples obtained from the trees were divided into two clades, which were designated Clade A and Clade B for convenience (Fig. 3, Suppl. material 8). In the COI gene reconstruction, Clade A was characterised by high support values based on both Bayesian analysis and the Maximum Likelihood method (PP = 1, BS = 100). Clade B was highly supported both in Bayesian analysis and the ML method (PP = 1, BS = 96). MOTU1–13 belong to Clade A, whereas MOTU14–20 belong to Clade B. Within Clade B, MOTU20 corresponds to *Pteraeolidia ianthina* (Angas, 1864), a valid species restricted to its type locality, Sydney Harbour, New South Wales (Wilson and Burghardt 2015); it was recovered as sister to MOTU14–19, which represent the *P. semperi* species complex. *Pteraeolidia ianthina* thus belongs to Clade B phylogenetically but is not part of the *P. semperi* complex and is kept separate from it throughout this study.

Notably, Clade B was recovered closer to the outgroup taxa rather than as sister to Clade A, the latter being placed on a long, isolated branch. This pattern is most plausibly an artefact of substitution saturation at deep nodes (see Discussion): the conservative COI of Clade B retains plesiomorphic similarity to the comparatively slowly evolving outgroups, whereas the highly accelerated COI of Clade A is saturated against all other lineages and lacks a stable deep placement. We therefore do not interpret the relative positions of Clade A, Clade B, and the outgroups along the COI backbone as reflecting true phylogenetic relationships.

At the same time, the *p*-distances between Clade A and Clade B in COI were above 30% (Suppl. material 5), which is abnormally high for the intrageneric *p*-distance.

A 16S tree was reconstructed but included far fewer terminals than the COI tree because 16S sequences are available for only a small number of specimens in GenBank; its topology was congruent with the COI tree (Suppl. material 9). The *p*-distances between these clades also exceeded 30% (Suppl. material 5).

For the tree based on H3, there was only one sequence in GenBank (accession number JQ997006.1), but the samples from Vietnam formed two clades with ten substitutions between them (Suppl. material 10). Clade A differed from the GenBank sequence JQ997006.1 by two substitutions. Pairwise *p*-distances between the two clades for the H3 gene were approximately 3% (Suppl. material 5).

#### Population structure

**Figure 4.**
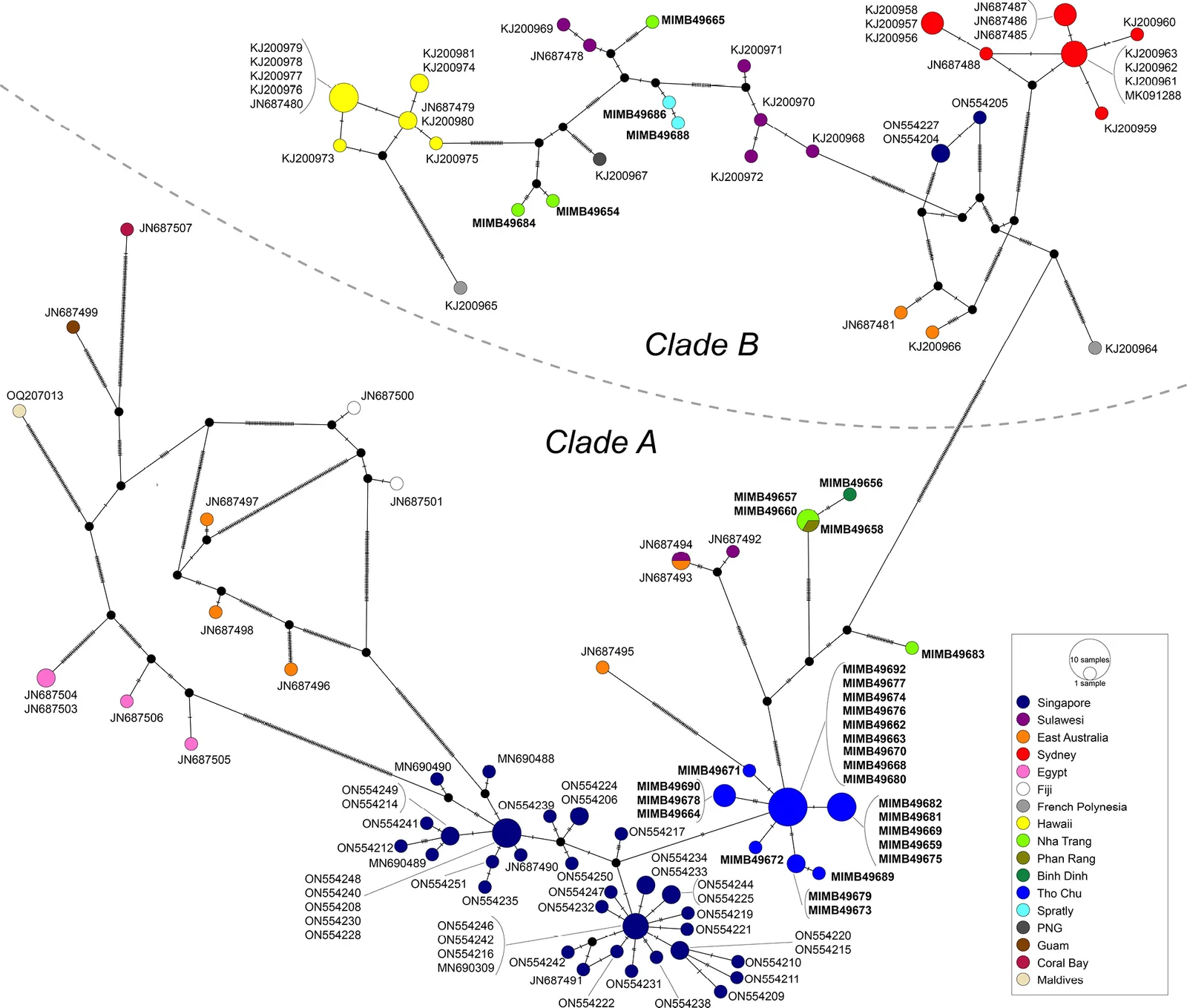
TCS haplotype network of the *Pteraeolidia semperi* species complex and *P. ianthina* based on COI sequences, constructed in PopART. Colours indicate the geographic origin of each haplotype, as shown in the legend. Circle size is proportional to the number of sequences sharing the same haplotype. Hatch marks on the connecting lines indicate the number of mutational steps between haplotypes or haplogroups. Sequences obtained in the present study are shown in bold. The dashed line separates Clade A from Clade B.

In the COI-based TCS haplotype network (Fig. 4), studied specimens formed two major groups. Overall, 24 haplogroups, based on collection localities and divergence data were identified. Clade A was represented by 13 haplogroups, Clade B by 11 haplogroups. Clade A included three haplogroups containing samples from Vietnam. The largest haplogroup comprised 22 samples from Vietnam (MIMB49659, MIMB49662–MIMB49664, MIMB49668–MIMB49682, MIMB49689, MIMB49690, MIMB49692) and 41 samples from Singapore (JN687490, JN687491, MN690309, MN690488–MN690490, ON554206, ON554208–ON554212, ON554214–ON554217, ON554219–ON554222, ON554224, ON554225, ON554228, ON554230–ON554235, ON554238–ON554242, ON554244, ON554246–ON554251). Samples from Vietnam and Singapore had different haplotypes, which differed at least by two substitutions. The closest haplogroup was differed by 29 substitutions and was represented by three samples (JN687493 from Moreton assigned to the East Australia geographical group, and JN687492 and JN687494 from Sulawesi). The other two haplogroups containing samples from Vietnam were represented by a small number of samples, all of which are from Vietnam. They differed from the largest haplogroup by 33 and 39 substitutions, respectively. The first was represented by four samples: two from Nha Trang (MIMB49657, MIMB49660), one from Phan Rang (MIMB49658), and one from Binh Dinh province (MIMB49656). The samples from Nha Trang and Phan Rang were genetically identical, while the sample from Binh Dinh differed from the others by two substitutions. The second haplogroup was represented by only one sample from Nha Trang (MIMB49683) and differed from the previously described haplogroup by 34 substitutions.

Clade B also contained three haplogroups represented by samples from Vietnam. The first haplogroup contained two samples from Nha Trang (MIMB49654 and MIMB49684). There were four substitutions between the two samples. The closest haplogroups to this haplogroup differed from it by 21 substitutions (a haplogroup represented by a single sample, KJ200967, from Papua New Guinea) or 24 substitutions (a haplogroup represented by 11 samples from Hawaii). One sample from Nha Trang (MIMB49665) was represented by a unique haplotype, differed from the closest haplogroup by ten substitutions. The latter haplogroup included two samples from Sulawesi (JN687478 and KJ200969) and two samples from the Spratly Archipelago (MIMB49686 and MIMB49688). The samples from the Spratly Islands differed from each other by one substitution and from the samples from Sulawesi by four substitutions.

### External morphology

#### General characteristics common to both clades (Figs 5, 6, 7)

Specimens of both clades possessed a slender body with a narrow foot and a relatively narrow, elongate head bearing rhinophores and oral tentacles, with head length approximately equal to rhinophore length. Numerous cerata were arranged on the dorsum in fan-shaped clusters, with the third pair of clusters usually bearing the highest number of cerata. Towards the posterior end of the body, both the size of cerata and their number per cluster decreased. The cerata at the posterior end of the body were not grouped and were conical in shape. Each cluster of cerata was attached to the dorsum by a common elevation. The rhinophores were club-shaped and perfoliate, with the basal part of the rachis tapering distally and lacking lamellae. Oral tentacles were smooth, conical, and 2–3 × longer than the rhinophores. The genital opening was large and muscular, located on the right side between the first and second ceratal clusters. The anus was cleioproctic.

**Figure 5.**
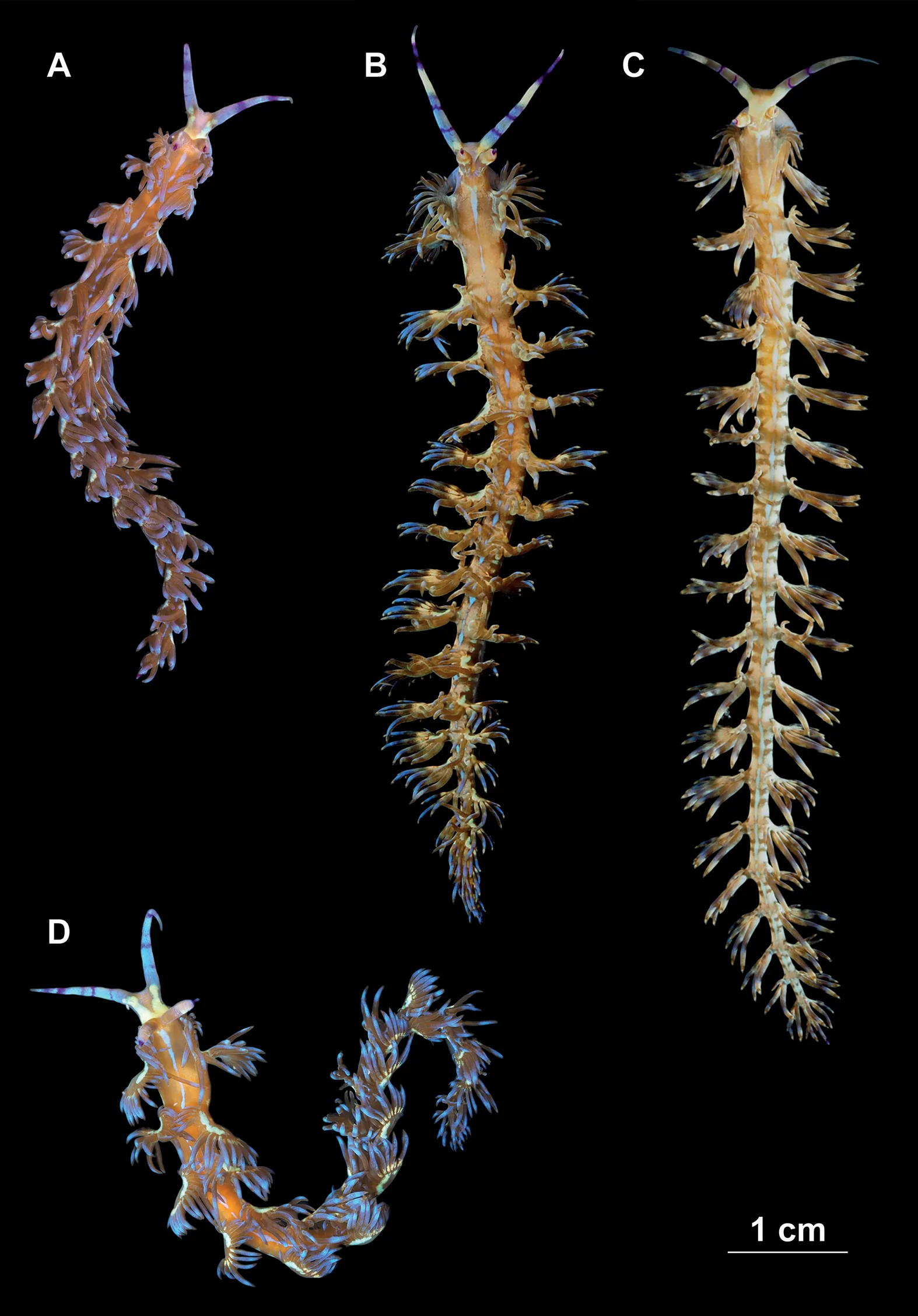
External morphology of *Pteraeolidia semperi* species complex, Clade A. **A**. MIMB 49667; **B**. MIMB 49671; **C**. MIMB 49659; **D**. MIMB 49680.

**Figure 6.**
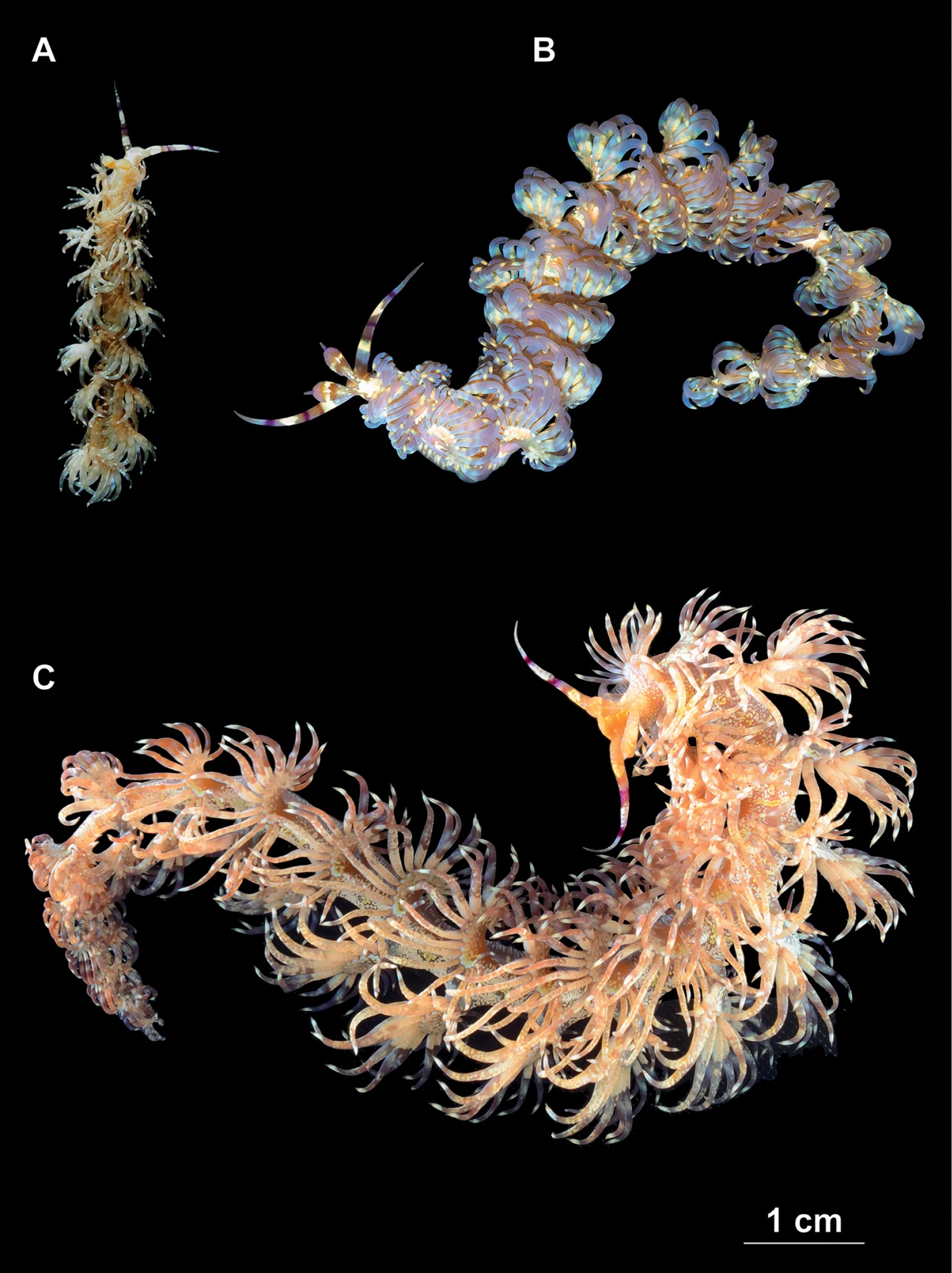
External morphology of *Pteraeolidia semperi* species complex, Clade B. **A**. MIMB 49688; **B**. MIMB 49686; **C**. MIMB 49684.

**Figure 7.**
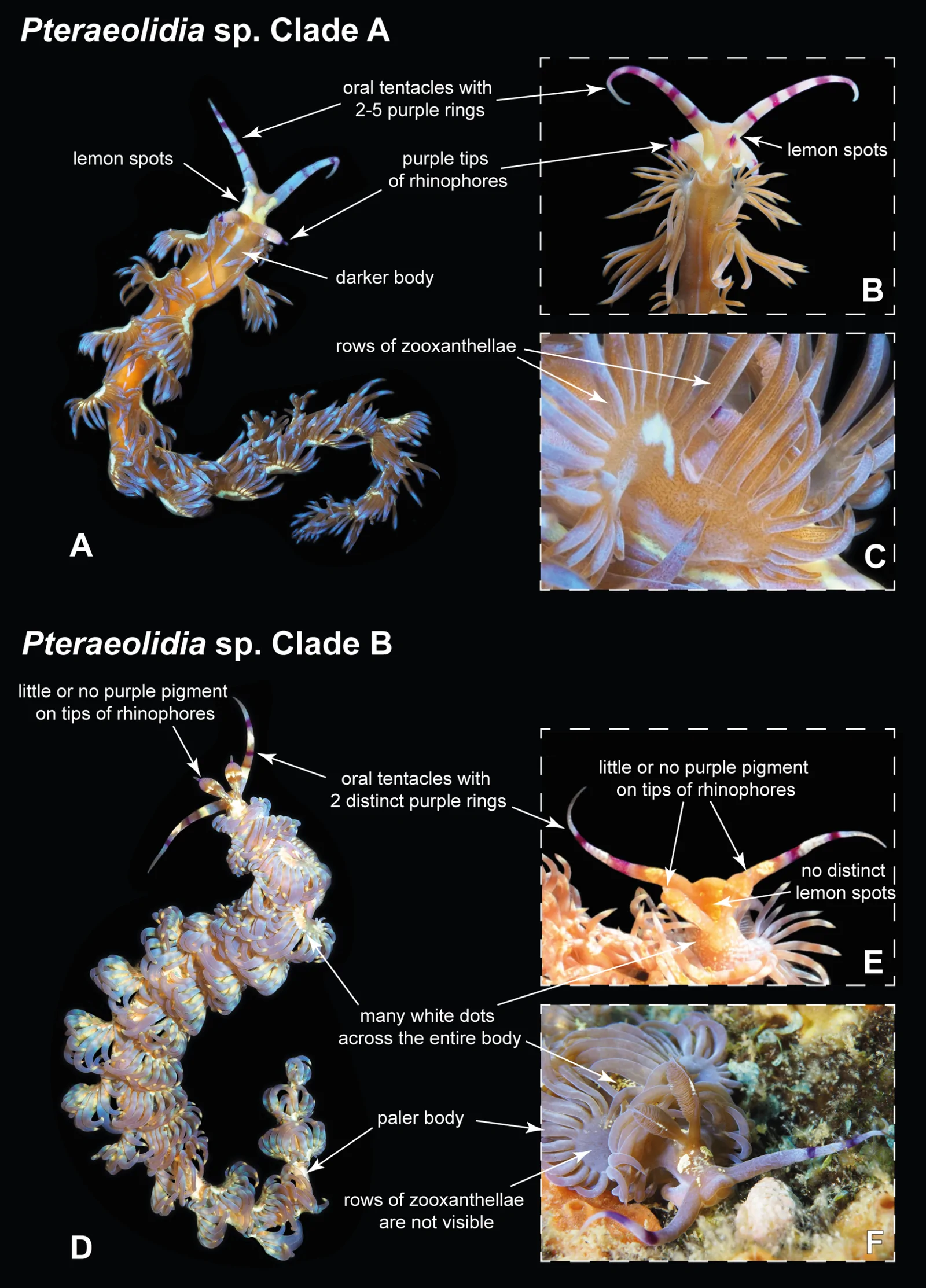
External morphology of representatives of the *Pteraeolidia semperi* species complex from the two major clades. **A**. Clade A, general body view, specimen MIMB 49680; **B**. Clade A, head in close-up view, specimen MIMB 49678; **C**. Clade A, cerata in close-up view, specimen MIMB 49666; **D**. Clade B, general body view, specimen MIMB 49686; **E**. Clade B, head in close-up view, specimen MIMB 49684; **F**. Clade B, cerata in close-up view, specimen MIMB 49654. Arrows indicate the main diagnostic external characters distinguishing specimens of the two clades.

Body length was highly variable and overlapped broadly between the two clades, ranging from ~10–100 mm in living specimens (one specimen reached 130 mm in life and 40 mm when preserved); it is therefore not diagnostic. Juveniles were considerably smaller, with body length of 4–10 mm.

#### Differences between clades

Clade A (Fig. 5): the number of ceratal clusters ranged from nine to 23 pairs; each cluster contained 7–21 cerata, which were arranged in a single row. Rhinophores possessed up to 38 lamellae. Juvenile specimens had body length of 4–10 mm, with cerata either not grouped or forming small clusters of 2–5 cerata. The number of ceratal rows in juveniles ranged from seven to 11, and rhinophores bore 6–17 lamellae. In the smallest juveniles, oral tentacles were approximately equal in length to the rhinophores, whereas in larger juveniles they were ~2 × as long. Clade B (Fig. 6): the number of ceratal clusters ranged from nine to 19 pairs; each cluster contained 7–53 cerata, which might be arranged in one or several rows. Rhinophores possessed up to 44 lamellae.

### Colouration

#### General characteristics common to both clades

The body colouration was generally variable and dependent on illumination. In the absence of or at low concentrations of symbionts, the body could be completely white or pale beige. A white or pale lemon stripe was frequently present along the midline of the dorsum between the ceratal clusters; it might remain visible in preserved specimens. The tips of cerata were often paler in the cnidosac region. The rhinophores were generally the same colour as the body. The lamellae were predominantly body-coloured, but several distal lamellae were partially pale, appearing as an upward-pointing wedge-shaped mark in lateral view. The oral tentacles bore bright rings, separated by intervals of semitransparent tissue.

#### Differences between clades

Clade A: the background colour of body was almost opaque and varied from pale beige to dark orange (Fig. 5). Body colouration was often nearly uniform, but in some individuals dark and pale transverse bands occurred across the dorsum. On the body sides below the cerata there were medium-sized oval spots that were white, bluish, or pale lemon-coloured, often alternating in orientation. The entire foot was uniformly coloured, but the end of tip was violet. The common elevations bearing cerata might carry a large white or pale lemon spot or was uniformly coloured. Cerata were almost opaque and usually of the same colour as the body, often slightly darker in the middle portion and paler with a bluish sheen in the distal third (Fig. 7C). The rhinophores are body-coloured along the lamellate region; immediately above the last lamellae the lamellae-free tip bears a band of violet pigmentation, while the extreme apex is again body-coloured (Fig. 7A, B). Oral tentacles always bear bright violet rings, which alternate with semitransparent regions (Fig. 7A, B). A single white ring was frequently present between the second and third violet rings (Fig. 5B, C). The rings were usually symmetrically arranged and 3–5 in number, more rarely fewer. On the dorsolateral sides of head there were often distinct pale lemon-coloured spots continuing from oral tentacles to rhinophores. In juveniles, the body was semitransparent and white, sometimes slightly unevenly brownish, with an opaque pale lemon spot on the head, a bright violet tip of the foot, violet tips of the rhinophores, and oral tentacles bearing only two violet rings with indistinct margins. Clade B: The body was semi-transparent, cream-violet, sometimes with a bluish hue, or beige-brown (Fig. 6). The dorsal surface of the body was covered with small to medium-sized round white or yellow pigment spots (Fig. 7D). Pigment spots of white or, more often, yellowish colour were present on the head, where they were symmetrically arranged, although the pattern differed among individuals. Both white and yellow spots might occur on the same specimen. On the sides of the body beneath the cerata, there were white or pale lemon-coloured spots ranging from very small and round to large vertically elongated patches with sharply defined margins that might occupy almost the entire lateral surface. One or two large spots were usually present between adjacent ceratal clusters. The lateral sides of the foot were finely lined with thin pale stripes. In some specimens, a large amount of white pigment was present near the bases of cerata. Cerata were semitransparent and generally of the same colour as body; they might bear small white pigment granules or be almost uniformly coloured (Fig. 7F). The rhinophores might bear white pigment granules. The tips of the rhinophores were most often body-coloured, but in rare cases might show violet pigmentation at lamellae-free tips (Fig. 7E, F). The oral tentacles were always body-coloured at the base and bore bright rings distally in the sequence white, violet, white, violet. Sometimes white pigment granules were present distal to the outer violet ring. Occasionally, immediately after the first white ring, a thin discontinuous diffuse violet ring was visible, much less distinct than the other violet rings. However, most commonly there were two clearly defined violet rings. More rarely, the white rings might be weakly expressed; in such cases the body colour from the base of the oral tentacle towards its tip gradually became paler.

### Internal morphology

**Figure 8.**
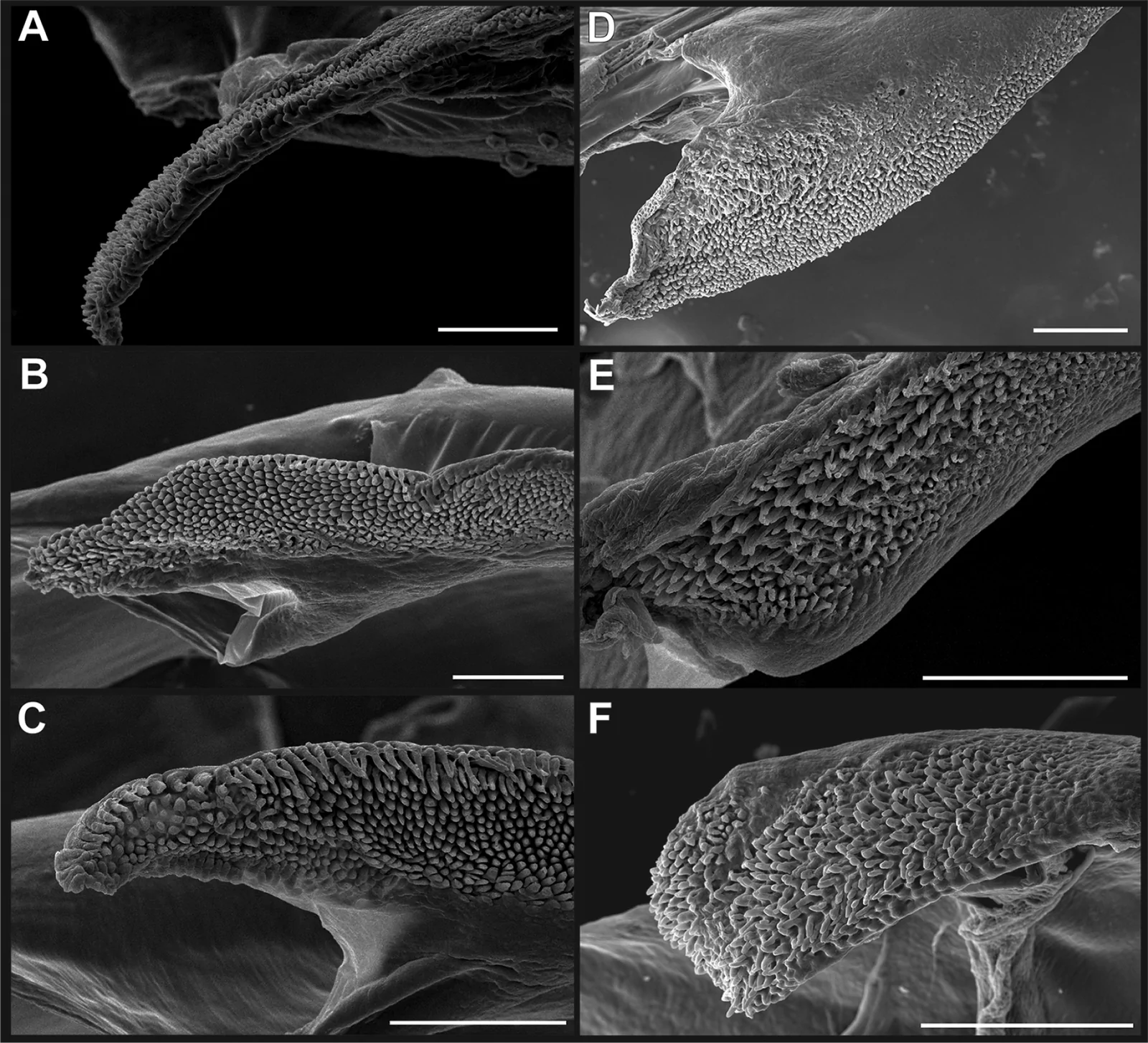
Jaw morphology of *Pteraeolidia semperi* species complex. **A–C**. Clade A; **D–F**. Clade B. **A**. MIMB 49656; **B**. MIMB 49668; **C**. MIMB 49683; **D**. MIMB 49654; **E**. MIMB 49665; **F**. MIMB 49686. Scale bars: 100 μm.

**Figure 9.**
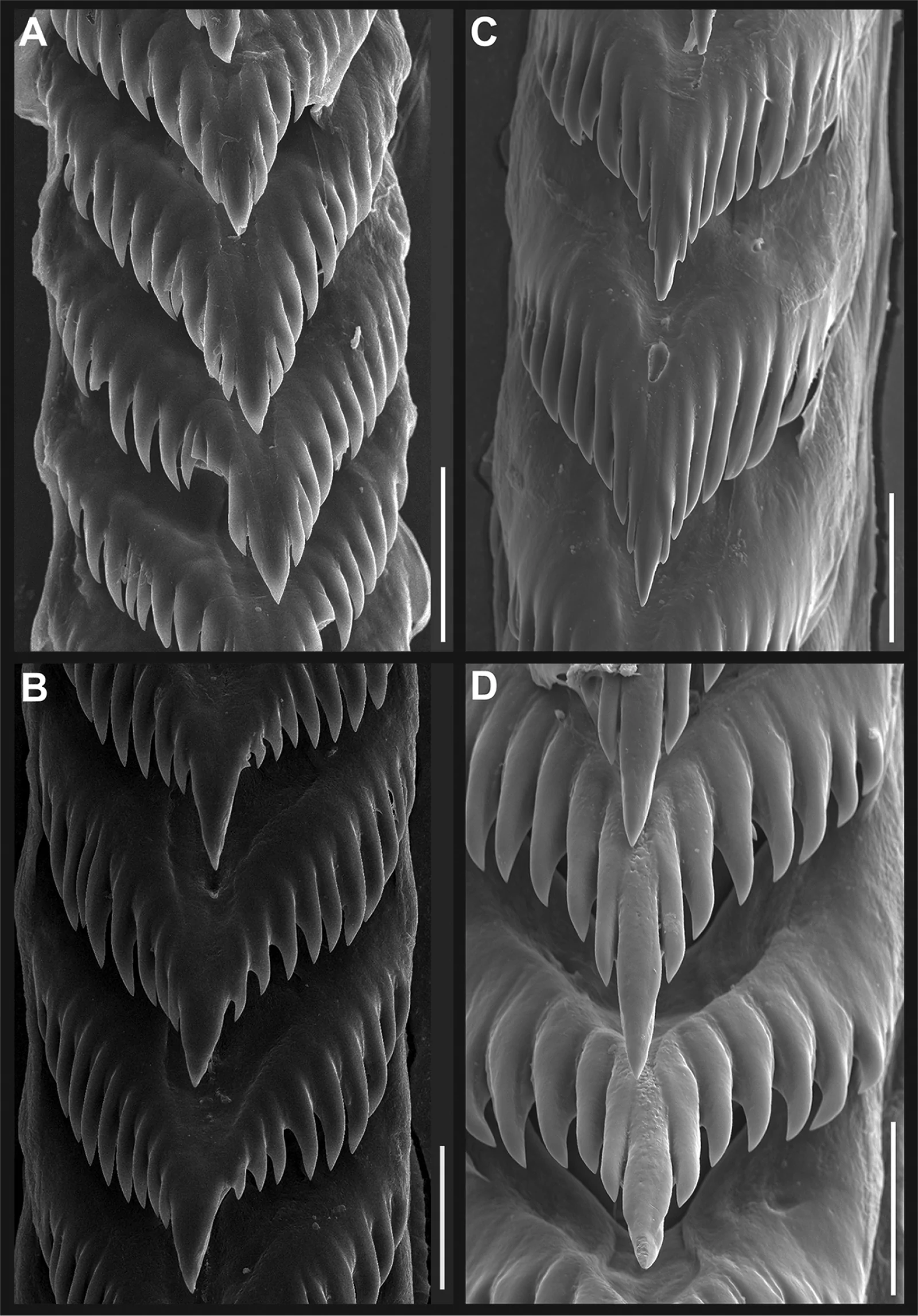
Radular morphology of *Pteraeolidia semperi* species complex. **A, B**. Clade A; **C, D**. Clade B. **A**. MIMB 49664; **B**. MIMB 49683; **C**. MIMB 49654; **D**. MIMB 49686. Scale bars: 50 μm.

The jaws are triangular, and the masticatory process bears multiple rows of long, rounded denticles (or in Clade B, flat rectangular denticles in the largest specimen) (Fig. 8). The radula is uniserial (Fig. 9). The number of radular rows in the examined specimens ranges from 17 to 26 in Clade A and 21 or 22 in Clade B. The teeth are triangular and bear an oval basal depression. The central cusp is not recessed, sharply pointed, and longer than the remaining cusps. On each side of the central cusp there are nine or ten lateral cusps; the cusps closest to the central one are relatively small and lack a separate base, arising directly from the central cusp. The reproductive system is diaulic. The ampulla is voluminous and strongly folded in a zigzag pattern. The vas deferens is thick and robust. The vagina is underdeveloped in the examined specimens.

## Discussion

### Systematics

The results of this study once more demonstrate that *Pteraeolidia semperi* represents a diverse species complex, which is in agreement with previous studies (Wilson and Burghardt 2015; Soon et al. 2023). Our morphological and molecular evidence suggests that the entire Indo-Pacific region is inhabited by distinct phylogenetic lineages (Fig. 3), of which taxonomical status requires further clarification. Most of these taxonomical units exhibit a restricted distribution in the Indo-West Pacific (Fig. 4), but in some localities, e.g. East Australia, Sulawesi, and Nha Trang (Vietnam), several genetically distinct populations were found. All this complicates the identification of the true *Pteraeolidia semperi*, described from the Philippines (Bergh 1870).

Our results show that *Pteraeolidia* in Vietnam coastal waters is represented by two diverged clades, named herein as Clade A and Clade B (Fig. 3). Representatives of Clade A are found in all surveyed water areas; representatives of Clade B are found in Nha Trang and Spratly only (Fig. 4). *Pteraeolidia* from Clade A are also found in Singapore, Sulawesi, Eastern Australia, Egypt, Fiji, Guam, Coral Bay, and Maldives, while representatives of Clade B, in addition to Nha Trang and Spratly, are found in Sulawesi, Papua New Guinea, Hawaii, Singapore, Eastern Australia, and French Polynesia. Specimens from Sydney, which also fall within Clade B, correspond to *P. ianthina* (MOTU20) and are treated here as a separate species rather than as part of the *P. semperi* complex. In Singapore, as in Vietnam, representatives of both clades are present (Soon et al. 2023). They are genetically close to the Vietnamese specimens and even belong to the same MOTU (MOTU1), which can be attributed to the close geographic proximity of these two regions (Fig. 1).

The clades recovered by Soon et al. (2023) correspond to our MOTUs as follows: their Singapore Clade A corresponds to MOTU1 (our Clade A); their Singapore Clade B to MOTU16 (our Clade B); *Pteraeolidia* ‘*semperi*’ A (Hawaii) to MOTU15 (Clade B); *Pteraeolidia* ‘*semperi*’ B (Indonesia) and C (PNG) both to MOTU14 (Clade B); *Pteraeolidia* ‘*semperi*’ D (GBR) to MOTU17 (Clade B); and *Pteraeolidia ianthina* (NSW) to our MOTU20 (Clade B).

At the same time, each clade is also characterised by a significant genetic intraclade divergence. According to the ASAP, several MOTUs from Clade A inhabit Vietnam (MOTU1, MOTU4, and MOTU5, Fig. 3). In the phylogeographic analysis, they are also distinguished into different haplogroups (Fig. 4), but no differences between them are found in the nuclear genome nor in morphological characters. This indicates a possible exchange of genes between representatives of the same clade (but different MOTUs). At the same time, representatives of Clades A and B show significant and consistent differences in histone H3 (Suppl. material 10), which indicates the absence of interbreeding between them. Thus, at least two species inhabit the waters of Vietnam, while the taxonomic status of individual MOTUs still needs to be clarified with the help of genomic data.

In addition to considerable genetic differences, Vietnamese representatives of Clade A and Clade B differ morphologically. The primary distinction between these species is their colouration patterns. Individuals of Clade A exhibit a darker pigmentation compared to those of Clade B. This phenomenon may be attributed to the distinct location of zooxanthellae in the body and cerata: in representatives of Clade A, rows of zooxanthellae are visible beneath the epithelium (Fig. 7C), whereas in representatives of Clade B they are not traceable, and only the brownish pigment derived from them is visible, suggesting that zooxanthellae in Clade B specimens are located deeper within the body tissues (Fig. 7F). This difference is apparent in adults of both clades and is independent of the ontogenetic colour change.

In addition, Clade B individuals frequently exhibit white pigment spots on dorsolateral sides of bodies (Figs 6C, 7D–F); however, this feature is not consistent in all examined specimens. Another distinguishing character is the presence of continuous lemon-coloured patches on the head that extend into the oral tentacles in members of Clade A (Fig. 7A, B). In members of Clade B, the patches, if present, are small and fragmented and do not extend into the oral tentacles (Fig. 7F). In representatives of Clade B, the cerata within each group are often arranged in several rows and appear bushier, whereas in Clade A they are arranged in a single row, resulting in a comparatively slenderer overall appearance of the animal. Furthermore, members of Clade A typically exhibit 3–5 purple rings on the oral tentacles (Fig. 7B), while members of Clade B commonly display two distinct purple rings (Fig. 7E).

The morphological characters distinguishing the two Vietnamese clades, together with their comparison to the original descriptions and to the redescriptions of Wilson and Burghardt (2015) of the available *Pteraeolidia* names, are summarised in Suppl. material 6. Both Vietnamese clades are also distinguishable in colouration from *Pteraeolidia ianthina* (MOTU20), which falls within Clade B but represents a separate species and has dark stripes along the edge of its cerata (Wilson and Burghardt 2015). However, no differences were found in internal morphology, both in the radular and jaw morphology and in the morphology of the reproductive system. Thus, representatives from Vietnam differ primarily in colouration and pigmentation pattern. The only non-chromatic external difference we observed concerns ceratal arrangement, a single row in Clade A versus several, bushier rows in Clade B, but this character is age-dependent and less pronounced in juveniles, and we therefore do not treat it as reliably diagnostic. Internal morphology, including the radula, jaws, and reproductive system, does not differ between the clades. The reliance on colouration is further complicated by the type material, since *Pteraeolidia semperi* was described from alcohol-bleached specimens, which makes direct comparison of colour with the type impossible.

#### High molecular divergence

At present, it is not possible to reliably estimate the number of species in the genus *Pteraeolidia*. The ASAP algorithm does not provide adequate resolution because a clear barcoding gap (i.e. a distinction between intra- and interspecific variation) is absent, and species boundaries therefore remain undefined. This is clearly reflected in analysis of pairwise *p*-distances (Suppl. material 5), as the level of divergence within a clade was found to be comparable to the interclade divergence: the maximum distance within Clade A in COI (27.7%) is very close to the minimum distance between Clade A and Clade B (27.9%) and likely a result of saturation.

Because COI is saturated at these depths, the deep nodes of the reconstruction retain little reliable signal. The conservative Clade B is consequently drawn towards the equally slowly evolving outgroups through shared ancestral (plesiomorphic) similarity, while the hyper-divergent Clade A, saturated against all other lineages, is placed on a long branch of unstable position. A closely comparable pattern was reported by Park and Poulin (2022) in an amphipod species complex, in which extreme COI divergence was concentrated in a single fast-evolving lineage and produced saturation, while nuclear markers showed far lower divergence, a discrepancy the authors likewise linked to the influence of microbial symbionts. Proximity in raw genetic distance therefore does not reflect true relationship here, and our recognition of Clade A and Clade B as distinct evolutionary lineages rests on the nuclear H3 data and on morphology rather than on the COI topology.

Differences among 16S sequences are at similarly high levels as those observed in COI. However, for the nuclear genome, *p*-distances, and numbers of substitutions between clades approach values that are typical for interspecific divergence in tropical aeolids (Ekimova et al. 2022). Thus, the abnormally high rate of evolution appears to be restricted to the mitochondrial genome. Such high mitochondrial divergence has already been observed in a recent study (Soon et al. 2023), who suggested that the phenomenon is associated with abnormally high mutation rates in the COI gene or is explained by poor taxon sampling in the phylogenetic reconstruction.

Elevated mitochondrial substitution rates and deep COI divergences have likewise been reported in other nudibranch lineages (Nimbs and Smith 2021; Layton and Wilson 2024), where they similarly complicate species delimitation. A comparable case occurs in another marine gastropod, the periwinkle *Melarhaphe neritoides*, in which mtDNA hyperdiversity is driven by an exceptionally high COI mutation rate (Fourdrilis et al. 2016, 2018). Such patterns are not restricted to molluscs: comparably deep COI divergences and rate-related artefacts in tree reconstruction have also been documented across several arthropod lineages (Kaltenpoth et al. 2012; Kovacs et al. 2024).

Here we show that such a high mutation rate occurs not only in the COI gene, but also in 16S, suggesting this is specific feature of the whole mtDNA of Clade A. It should also be noted that intraclade divergence within Clade A is also much higher than that observed within Clade B (Fig. 3). We hypothesise that the marked mitochondrial divergence of Clade A may be a result of a high evolutionary rate of mtDNA due to symbiosis with zooxanthellae. This hypothesis comes from the idea that the presence of photosymbiosis may facilitate speciation resulting in a greater species diversity (Wägele 2004). For example, the genus *Phyllodesmium*, which is known by its symbiotic association with zooxanthellae, harbours a significantly higher species diversity compared to its closely related sister genera, for which symbiosis with algae is not characteristic (Wägele et al. 2010).

Mitochondria are directly implicated in respiration, and their genome encompasses genes of respiration-related enzymes. It is likely that these genes may be under natural selection pressures in conditions of a close and specific photosymbiotic system between the nudibranchs and their algae, and therefore these markers cannot be used for phylogenetic purposes (Edwards 2009). Another possible explanation is that high oxygen levels in tissues of *Pteraeolidia* lead to elevated concentrations of reactive oxygen, which can affect the reparation systems and increase mutagenesis (Waris and Ahsan 2006). The second hypothesis better explains the presence of mutations not only in genes encoding respiratory enzymes, but also in ribosomal RNA genes. However, these hypotheses are preliminary, and further genomic studies are required to reveal the nature of this unique phenomenon.

#### Biogeography

Almost all the haplogroups present in the haplotype network exhibit high geographical structure. This structure is evident in haplogroups from the following territories: Egypt, Fiji, Singapore and Tho Chu, Hawaii, and Sydney, the last of these being *P. ianthina* (Fig. 4). However, in some cases we identified the presence of different haplogroups in a single region: these include the samples from Sulawesi, East Australia, Singapore, and Nha Trang.

Samples from Vietnam show a different geographical connectivity, depending on the collection site. Samples from marginal areas, like Tho Chu and the Spratly Islands, are close to those from neighbouring regions: samples from Tho Chu are close to those from Singapore, and samples from Spratly are close to several haplotypes found in Sulawesi (Fig. 4). The Tho Chu group of islands is located in the Gulf of Thailand and is much closer to Singapore than other collection sites. The Spratly archipelago is the most distant of the collection sites to the east and is close to the Philippines and Sulawesi. Samples from central Vietnam are highly diverse. Individuals collected in Nha Trang belong to both clades and exhibit a high number of substitutions between each other (Fig. 4).

Our work reveals a pattern similar to the idea of the Coral Triangle as a centre of marine biodiversity (Fitzpatrick et al. 2011; Fritts-Penniman et al. 2020). The most genetically diverse samples are found in Sulawesi, located at the heart of the Coral Triangle, and from nearby Vietnamese waters. The same was observed in the East Australia waters: as this area is also located near the Coral Triangle, high sample diversity can be expected there.

Conversely, samples from more peripheral areas are represented by a single haplogroup in each case and have low nucleotide diversity, such as Hawaii, Sydney, Egypt, and Fiji. Hawaii and Egypt are the most distant territories from the Central West Pacific and samples from these locations show the highest *p*-distances from other samples (Fig. 4, Suppl. material 5). The most surprising region is Sydney, as samples from there form an apparently isolated group that differs genetically from other haplogroups in East Australia by a large number of substitutions. This haplogroup corresponds to the species *Pteraeolidia ianthina* (Wilson and Burghardt 2015).

As GenBank data on samples from various Indo-Pacific regions mainly includes mitochondrial gene sequences, it is not possible to accurately determine the number of species based on their geographical distribution and identify a possible geneflow between distant areas. To formulate a species hypothesis, it is necessary to use rapidly evolving nuclear genes. When used in conjunction with other data, these will help us to understand the systematics of this genus.

In conclusion, our integrative analysis of the *Pteraeolidia semperi* species complex in Vietnam, combining mitochondrial and nuclear markers with morphological data, reveals two well-supported clades. Species boundaries are best resolved by combining fixed diagnostic substitutions in the nuclear H3 gene with MOTU delimitation of COI, whereas morphological differentiation between the clades is predominantly chromatic, since ceratal arrangement is age-dependent and unreliable as a diagnostic character. The exceptionally high mitochondrial divergence recovered within this complex, particularly in Clade A, is consistent with the accelerated COI evolution documented in other nudibranchs and marine gastropods, and should therefore be interpreted with caution in phylogenetic reconstruction. Our results underscore the value of an integrative, multi-marker approach for delimiting species in groups where mitochondrial data alone may be misleading.

## Additional information

### Artificial Intelligence (AI) use

The authors accept full responsibility for the content of the manuscript, including the disclosure of any use of AI.

No AI tools were used in the preparation of this manuscript.

### Author ORCIDs

M.N. Meshik https://orcid.org/0009-0005-7237-054X

T.I. Antokhina https://orcid.org/0000-0002-0857-4186

Yu.V. Deart https://orcid.org/0000-0001-6847-8295

O.V. Chichvarkhina https://orcid.org/0009-0001-4737-4865

I.A. Ekimova https://orcid.org/0000-0002-1846-0780

### Data availability

All of the data that support the findings of this study are available in the main text or Supplementary Information.

## Supplementary materials

Supplementary material 1 Material examined Data type xlsx Explanation note This file provides specimen-level data for the material examined in the present study, including voucher numbers, clade and MOTU assignment, sampling locality, depth, habitat, geographic coordinates, collection date, and collector https://binary.pensoft.net/file/1796045This dataset is made available under the Open Database License (http://opendatacommons.org/licenses/odbl/1.0/). The Open Database License (ODbL) is a license agreement intended to allow users to freely share, modify, and use this Dataset while maintaining this same freedom for others, provided that the original source and author(s) are credited. Maria N. Meshik, Tatiana I. Antokhina, Yury V. Deart, Olga V. Chichvarkhina, Irina A. Ekimova

Supplementary material 2 Primers and PCR conditions used in the present study Data type docx Explanation note This file contains the primers and PCR conditions used in the present study for amplification of the COI, 16S rRNA, and H3 markers, together with the corresponding references. https://binary.pensoft.net/file/1796046This dataset is made available under the Open Database License (http://opendatacommons.org/licenses/odbl/1.0/). The Open Database License (ODbL) is a license agreement intended to allow users to freely share, modify, and use this Dataset while maintaining this same freedom for others, provided that the original source and author(s) are credited. Maria N. Meshik, Tatiana I. Antokhina, Yury V. Deart, Olga V. Chichvarkhina, Irina A. Ekimova

Supplementary material 3 GenBank sequences used in the present study Data type xlsx Explanation note This file lists the GenBank sequences used in the present study, together with museum voucher numbers, taxonomic identification, sampling localities, and references to the original publications. https://binary.pensoft.net/file/1796047This dataset is made available under the Open Database License (http://opendatacommons.org/licenses/odbl/1.0/). The Open Database License (ODbL) is a license agreement intended to allow users to freely share, modify, and use this Dataset while maintaining this same freedom for others, provided that the original source and author(s) are credited. Maria N. Meshik, Tatiana I. Antokhina, Yury V. Deart, Olga V. Chichvarkhina, Irina A. Ekimova

Supplementary material 4 Correspondence of COI-based MOTUs between newly generated and GenBank sequences Data type xlsx Explanation note This file shows the correspondence of molecular operational taxonomic units (MOTUs) inferred from the COI phylogenetic tree between newly generated sequences and sequences retrieved from GenBank. https://binary.pensoft.net/file/1796048This dataset is made available under the Open Database License (http://opendatacommons.org/licenses/odbl/1.0/). The Open Database License (ODbL) is a license agreement intended to allow users to freely share, modify, and use this Dataset while maintaining this same freedom for others, provided that the original source and author(s) are credited. Maria N. Meshik, Tatiana I. Antokhina, Yury V. Deart, Olga V. Chichvarkhina, Irina A. Ekimova

Supplementary material 5 Pairwise *p*-distances between MOTUs based on COI, 16S rRNA, and H3 sequences Data type xlsx Explanation note This file contains pairwise p-distances between molecular operational taxonomic units (MOTUs) calculated for the COI, 16S rRNA, and H3 markers used in the present study. https://binary.pensoft.net/file/1796049This dataset is made available under the Open Database License (http://opendatacommons.org/licenses/odbl/1.0/). The Open Database License (ODbL) is a license agreement intended to allow users to freely share, modify, and use this Dataset while maintaining this same freedom for others, provided that the original source and author(s) are credited. Maria N. Meshik, Tatiana I. Antokhina, Yury V. Deart, Olga V. Chichvarkhina, Irina A. Ekimova

Supplementary material 6 Comparison of external, chromatic and radular characters among the two Vietnamese clades and the described species of *Pteraeolidia* Data type xlsx Explanation note This file provides a comparison of the principal external, chromatic and radular morphological characters of the two Vietnamese clades (Clade A and Clade B) of the *Pteraeolidia semperi* species complex with the original descriptions of the nominal species (*Pteraeolidia ianthina*, *P. semperi*, *P. scolopendrella*, and *P. annulata*) and with the redescriptions of *P. ianthina* and *P. semperi* provided by Wilson and Burghardt (2015). https://binary.pensoft.net/file/1796050This dataset is made available under the Open Database License (http://opendatacommons.org/licenses/odbl/1.0/). The Open Database License (ODbL) is a license agreement intended to allow users to freely share, modify, and use this Dataset while maintaining this same freedom for others, provided that the original source and author(s) are credited. Maria N. Meshik, Tatiana I. Antokhina, Yury V. Deart, Olga V. Chichvarkhina, Irina A. Ekimova

Supplementary material 7 ASAP species partitioning of the COI dataset under three distance models (Jukes–Cantor [JC69], Kimura 2-parameter [K80], and simple pairwise *P*-distances; columns) for the complete dataset (Total) and for Clade A and Clade B analysed separately (rows). For each analysis the ten best-scoring partitions are shown with their number of subsets and ASAP scores; coloured bars indicate MOTU assignment Data type pdf Explanation note This file contains the ASAP species-partitioning results for the COI dataset of the *Pteraeolidia semperi* species complex and *P. ianthina*, obtained under three distance models (Jukes–Cantor [JC69], Kimura 2-parameter [K80], and simple pairwise P-distances) for the complete dataset (Total) and for Clade A and Clade B analysed separately. For each analysis the ten best-scoring partitions are listed with their number of subsets and ASAP scores, and MOTU assignment is indicated by coloured bars. https://binary.pensoft.net/file/1796051This dataset is made available under the Open Database License (http://opendatacommons.org/licenses/odbl/1.0/). The Open Database License (ODbL) is a license agreement intended to allow users to freely share, modify, and use this Dataset while maintaining this same freedom for others, provided that the original source and author(s) are credited. Maria N. Meshik, Tatiana I. Antokhina, Yury V. Deart, Olga V. Chichvarkhina, Irina A. Ekimova

Supplementary material 8 Maximum likelihood tree based on COI sequences of the *Pteraeolidia semperi* species complex and *P. ianthina* Data type tif Explanation note This file contains the phylogenetic tree of the *Pteraeolidia semperi* species complex and *P. ianthina* based on COI sequences. Specimens are assigned to molecular operational taxonomic units (MOTUs), and the figure illustrates the phylogenetic structure of the analysed material. Geographic distribution is indicated by colour coding, with the corresponding regions shown in the legend below. https://binary.pensoft.net/file/1796052This dataset is made available under the Open Database License (http://opendatacommons.org/licenses/odbl/1.0/). The Open Database License (ODbL) is a license agreement intended to allow users to freely share, modify, and use this Dataset while maintaining this same freedom for others, provided that the original source and author(s) are credited. Maria N. Meshik, Tatiana I. Antokhina, Yury V. Deart, Olga V. Chichvarkhina, Irina A. Ekimova

Supplementary material 9 16S-based phylogenetic tree of the *Pteraeolidia semperi* species complex and *P. ianthina* Data type tif Explanation note This file contains the phylogenetic tree of the *Pteraeolidia semperi* species complex and *P. ianthina* based on 16S sequences, showing the separation of the analysed specimens into Clade A and Clade B. Specimens generated in the present study are highlighted in bold, and the outgroup taxa are indicated at the base of the tree. https://binary.pensoft.net/file/1796053This dataset is made available under the Open Database License (http://opendatacommons.org/licenses/odbl/1.0/). The Open Database License (ODbL) is a license agreement intended to allow users to freely share, modify, and use this Dataset while maintaining this same freedom for others, provided that the original source and author(s) are credited. Maria N. Meshik, Tatiana I. Antokhina, Yury V. Deart, Olga V. Chichvarkhina, Irina A. Ekimova

Supplementary material 10 H3-based phylogenetic tree of the *Pteraeolidia semperi* species complex Data type tif Explanation note This file contains the phylogenetic tree of the *Pteraeolidia semperi* species complex based on H3 sequences, showing the separation of the analysed specimens into Clade A and Clade B. https://binary.pensoft.net/file/1796054This dataset is made available under the Open Database License (http://opendatacommons.org/licenses/odbl/1.0/). The Open Database License (ODbL) is a license agreement intended to allow users to freely share, modify, and use this Dataset while maintaining this same freedom for others, provided that the original source and author(s) are credited. Maria N. Meshik, Tatiana I. Antokhina, Yury V. Deart, Olga V. Chichvarkhina, Irina A. Ekimova

Supplementary material 11 Bayesian and maximum likelihood phylogenetic trees Data type zip Explanation note This file (compressed archive) contains the Bayesian and maximum likelihood (ML) trees inferred for the COI, 16S rRNA, and H3 markers, together with a table linking the working specimen codes to the corresponding MIMB museum voucher numbers. https://binary.pensoft.net/file/1796055This dataset is made available under the Open Database License (http://opendatacommons.org/licenses/odbl/1.0/). The Open Database License (ODbL) is a license agreement intended to allow users to freely share, modify, and use this Dataset while maintaining this same freedom for others, provided that the original source and author(s) are credited. Maria N. Meshik, Tatiana I. Antokhina, Yury V. Deart, Olga V. Chichvarkhina, Irina A. Ekimova
