# Supplementary material for: Dragons guard secrets: integrative systematics of Pteraeolidia semperi species complex (Gastropoda, Nudibranchia) from Vietnam
Source: Zookeys. 2026 Oct 2;1293:347–76. doi: 10.3897/zookeys.1293.195366 (PMC13653254; doi:10.3897/zookeys.1293.195366)
Supplement: Supplementary file 1 [file zookeys-1293-347_article-195366__-s007.pdf]

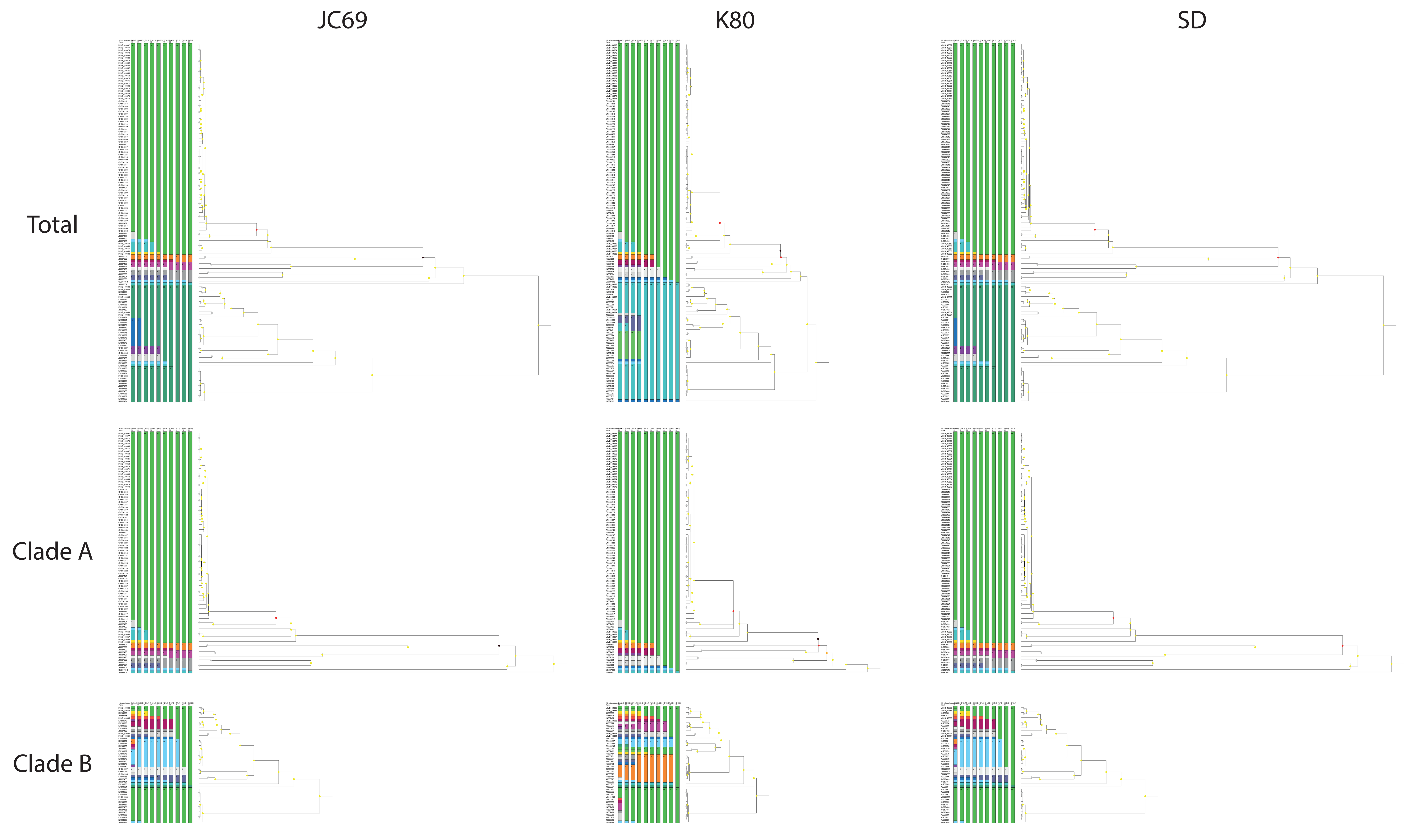

JC69
K80
SD
Total
Clade A
Clade B
