# Supplementary material for: Dragons guard secrets: integrative systematics of Pteraeolidia semperi species complex (Gastropoda, Nudibranchia) from Vietnam
Source: Zookeys. 2026 Oct 2;1293:347–76. doi: 10.3897/zookeys.1293.195366 (PMC13653254; doi:10.3897/zookeys.1293.195366)
Supplement: Supplementary file 2 [file zookeys-1293-347_article-195366__-s002.docx]

| Marker | Primers | PCR conditions | Reference |
| --- | --- | --- | --- |
| Cytochrome c oxidase subunit I | **LCO1490**  GGT CAA CAA ATC ATA AAG ATA TTG G  **HCO2198**  TAA ACT TCA GGG TGA CCA AAA AAT CA | 5 min - 94°C, 35×[15 s - 95°C, 45 s - 45°C, 1 min - 72°C], 7 min - 72°C | Folmer et al. 1994 |
| 16S rRNA | **16Sar-L**  CGC CTG TTT ATC AAA AAC AT  **16S R**  CCG RTY TGA ACT CAG CTC ACG | 5 min - 94°C, 35×[20 s - 95°C, 30 s - 52°C, 45 s - 72°C], 7 min - 72°C | Palumbi et al. 1991 |
| Histone H3 | **H3AF**  ATG GCT CGT ACC AAG CAG ACV GC  **H3AR**  ATA TCC TTR GGC ATR ATR GTG AC | 5 min - 94°C, 35×[15 s - 94°C, 30 s - 50°C, 45 s - 72°C], 7 min - 72°C | Colgan et al. 1998 |

1. Colgan DJ, McLauchlan A, Wilson GD, Livingston S, Edgecombe G, Macaranas J, Cassis G, Gray M (1999) Histone H3 and U2 snRNA DNA sequences and arthropod molecular evolution. Australian Journal of Zoology 46: 419–437. https://doi.org/10.1071/ZO98048
2. Folmer O, Hoeh W, Black M, Vrijenhoek R (1994) Conserved primers for PCR amplification of mitochondrial DNA from different invertebrate phyla. Molecular Marine Biology and Biotechnology 3: 294–299.
3. Palumbi SR, Martin AP, McMillan WO, Romano S, Stice L, Grabowsky G (1991) The simple fool’s guide to PCR.
